# Chrysophanol attenuates cognitive impairment, neuroinflammation, and oxidative stress by TLR4/NFκB-Nrf2/HO-1 and BDNF/VEGF signaling in stress-intensified PTZ induced epilepsy in mice

**DOI:** 10.3389/fphar.2024.1446304

**Published:** 2024-11-22

**Authors:** Jehan Zeb Khan, Syeda Rida Zainab, Mujeeb Ur Rehman, Muhammad Abid, Fawad Ali Shah, Najeeb Ur Rehman, Muhammad Khalid Tipu

**Affiliations:** ^1^ Department of Pharmacy, Faculty of Biological Sciences, Quaid-i-Azam University, Islamabad, Pakistan; ^2^ Department of Pharmacy, Iqra University, Islamabad, Pakistan; ^3^ Swat College of Pharmaceutical Sciences, Swat, Khyber Pakhtunkhwa, Pakistan; ^4^ Department of Pharmacology and Toxicology, College of Pharmacy, Prince Sattam Bin Abdulaziz University Al-Kharj, Al-Kharj, Saudi Arabia

**Keywords:** chrysophanol, stress, pentylenetetrazole, epilepsy, neuroinflammation, oxidative stress, neurodegeneration

## Abstract

**Background:**

Stress is among the most common comorbid conditions with epilepsy and a strong factor in the pathophysiology of seizures. An imbalance in neuronal circuits causes recurrent unprovoked seizures in epilepsy. Dysregulation of BDNF/VEGF expression, oxidative stress, increased levels of neuroinflammatory cytokines, and increased expression of apoptotic genes contribute to the underlying cause of the seizure.

**Objectives:**

Chrysophanol, an anthraquinone, has broad-spectrum therapeutic potential. This study evaluated the neuroprotective effect of chrysophanol with underlying pathways in PTZ-induced epilepsy with stress as a comorbid condition.

**Methods:**

Male mice were given 35 mg/kg of PTZ every other day to induce seizures. In addition, they were exposed to 120 min of daily restraint stress for 21 days to induce stress. Chrysophanol (0.1, 1, 10 mg/kg) was administered to the mice 30 min before the PTZ in the acute study. The most effective dose (10 mg/kg) was proceeded for the chronic epilepsy model. Following this, various tests were conducted, including behavioral assessments for memory impairment and stress, analysis of antioxidant levels, histopathological and immunohistochemistry examinations, measurement of cortisol levels using ELISA, and gene expression analysis using RT-PCR.

**Results:**

Chrysophanol demonstrated a notable decrease in both the intensity and frequency of seizures. Additionally, it effectively boosted the levels of important antioxidants such as GSH, GST, and CAT, while simultaneously reducing the levels of MDA and Nitric oxide. The histopathological analysis also showed improvement in overall morphology and survival of neurons. Chrysophanol treatment effectively showed an increase in the expression of BCL-2, and Nrf-2 with a decrease in BAX expression confirmed by immunohistochemistry. Dysregulation of vascular permeability factor, production of inflammatory cytokines, and apoptotic gene expression was successfully reversed after chrysophanol treatment analyzed through RT-PCR. Cortisol concentration was decreased in treatment groups analyzed through Enzyme-linked immunoassay. Molecular docking of chrysophanol with different proteins declared the binding affinity of the ligands with the target sites of proteins.

**Conclusion:**

In conclusion, chrysophanol demonstrated remarkable neuroprotective and antiepileptic effects at a dose of 10 mg/kg in stress-exacerbated PTZ-induced epilepsy following the TLR4/NFκB -Nrf2/HO-1 and BDNF/VEGF pathways.

## 1 Introduction

Epilepsy is a chronic neurological disease that manifests symptoms in the form of seizures that are periodic and recurrent and are caused by electrical disturbances in the brain (Javaid et al., 2024). This activity leads to temporary disruptions in normal brain function. Seizures can manifest in various forms ranging from subtle moments of altered consciousness or behavior to dramatic convulsion (Amin et al., 2022). Epilepsy is a major global health issue, impacting around 50 million individuals worldwide (Dehkordi et al., 2023). Its prevalence varies by region, age, and other factors. Generally, the prevalence of epilepsy is estimated to be around 1% of the population globally (Espinosa-Garcia et al., 2021; Perveen et al., 2024). To comprehend the mechanisms and factors involved in epilepsy, one must delve into the intricate workings of the brain’s electrical circuits, the underlying causes of abnormal activity, and the various factors that can influence the development and manifestation of seizures. Genetically, an imbalance at either the circuit or receptor level or an abnormal function of ionic channels can also produce seizures (Wu et al., 2018). Mechanistically, several pathways are involved in active seizure, such as hyperexcitation of the glutaminergic system, decline of the GABAergic system, and alterations in the metabolic system. Additionally, several neuromodulators play an integral part in the occurrence of epileptic seizures (Javaid et al., 2023). Research has shown that the increase in vascular endothelial growth factor (VEGF) and decrease in brain-derived neurotrophic factor (BDNF) levels in the brain are linked to the development of epilepsy. The toll-like receptor 4 (TLR4) pathway, a key player in inflammation, triggers the formation of inflammatory cytokines such as nuclear factor kappa B (NFκB), tumor necrosis factor (TNF-α), interleukin-1 (IL-1β), and, which play a significant role in the production of seizures. Epileptic episodes can range from brief moments of attention deficit and muscle jerks to prolonged convulsions that significantly affect the patient’s quality of life. The frequency of seizures can vary greatly, ranging from less than one attack per year to several per day. Stress is one of the major triggers of epilepsy. It can affect the growing brain and contribute to the development of epilepsy. In the adult brain, persistent stress can cause changes in brain function, leading to the development of seizures or epilepsy (Espinosa-Garcia et al., 2021; Privitera et al., 2014). Studies conducted on animals indicate that being exposed to stressors for a long period and an increase in cortisol levels in the blood can lead to depressive and anxiety-like behavior in animals, as well as increase the risk of seizures (Espinosa-Garcia et al., 2021; Kotwas et al., 2017). Stress and depression are common psychiatric comorbidities that affect approximately 62% of epileptic patients. However, they often go unrecognized. Both conditions are linked to neuroinflammation and the production of oxidative stress markers (Canzian et al., 2021; Günes et al., 2020). Studies have shown that epileptic seizures cause a deficit in learning and spatial memory (Nkwingwa et al., 2023). Stress itself is related to compromised memory and learning behavior in mice (Khan et al., 2024). Numerous studies have demonstrated the crucial role of oxidative stress in the development of epilepsy (Pereira et al., 2012). Depletion of antioxidants within the brain such as Glutathione S-transferase (GSH), Reduced glutathione (GST), and catalase (CAT), and a significant rise in reactive oxygen species (ROS) such as Malonaldehyde (MDA) and Nitric oxide (NO) disturb normal physiology. This, in turn, causes oxidative stress, inflammation, and apoptosis (Yilgor and Demir, 2024; Ahmed Amar et al., 2019). Conventional anti-epileptic treatment has several side effects including fatigue, gastric issues, blurry vision, and decline in concentration and memory.

Natural products serve as crucial sources for obtaining therapeutic agents, encompassing both primary and secondary metabolites (Mushtaq et al., 2018; Dias et al., 2012; Gurnani et al., 2014). They can be effectively utilized in various disorders due to their multitargeted nature and can be a better alternative for the management of epileptogenesis as well (Schmidt, 2009; Prateeksha et al., 2019; Wangchuk, 2018). Anthraquinones are the secondary metabolites produced by various plants and have been used for their therapeutic activities (Singh et al., 2017; Xie et al., 2019). Chrysophanol is an anthraquinone with broad-spectrum therapeutic potential and has been used in conventional Chinese and Korean medicinal systems due to its therapeutic effects on human health. It is a 1,8- dihydroxy-3- methyl derivative of the 9, 10- anthracene dione ring (Malik et al., 2010; Diaz-Munoz et al., 2018). Over the years, several beneficial properties of chrysophanol have been evaluated including its anti-cancer, anti-viral, anti-diabetic anti-inflammatory, anti-obesity, and neuroprotective (Lo et al., 2012; Lin et al., 2015). Chrysophanol is a tricyclic aromatic quinone and has been found in plants and animals as well as in the microbial world. Rumex and Rheum, which belong to the Polygonaceae family, are the two important sources of chrysophanol (Zhang et al., 2014; Singh et al., 2017). Because of its structural relationship, chrysophanol has diverse biological activities including neuroprotective activity (Zhang et al., 2014). Due to its diverse biological activities especially anti-inflammatory activity, it might be a useful agent in the treatment of epilepsy due to its structural activity relationship. Chrysophanol is evaluated for its potential neuroprotective and anti-inflammatory activity in stressed intensified PTZ kindled mice. After that, a series of tests were carried out. These included evaluating behavior to assess memory impairment and stress, analyzing antioxidant levels, conducting histopathological and immunohistochemistry examinations, measuring cortisol levels using ELISA, and analyzing gene expression using RT-PCR.

## 2 Material and methods

### 2.1 Animals

For this experiment, BALB/c male mice weighing approximately 25-30g were procured from the National Institute of Health Sciences in Islamabad. Mice were acclimatized for 1 week before the start of the experiment. All animals were housed in the animal facility at Quaid I Azam University, Islamabad. Mice were kept in plastic cages that had sawdust bedding, sterile water, and rodent food *ad libitum*. To prevent overcrowding only 4 mice were placed in each cage. Mice were housed in a favorable environment with a 12-h light-dark cycle and a constant temperature of 23°C ± 2°C. All *in vivo* and behavioral tests were conducted during the light cycle (Approval No. #BEC-FBS-QAU2023-560).

### 2.2 Drugs and chemicals

Dimethylsulfoxide (DMSO) (Sigma Aldrich), 0.9% normal saline, Pentylenetetrazol (Sigma Aldrich), chrysophanol (Angene chemical), Diazepam (Valium^®^; Batch No F2002), Formaldehyde, Hydrogen peroxide (H_2_O_2_), Catalase (CAT), Griess reagent, trichloroacetic acid-II, N-(1-naphthyl)ethylenediamine dihydrochloride, 1-chloro-2,4-dinitrobenzene, Ethanol, 5,5′- dithio-bis-(2-nitrobenzoic acid), Glutathione S-transferase (GST), Reduced glutathion (GSH), Xylene, and Hematoxylin and Eosin (H&E) were purchased from Sigma-Alrich, St. Louis, MO, United States. Primary antibiotics including TNF-α, IL-1β, and NF-κB have corresponding catalogs numbers SC-365949, SC-52746, SC-52012, and SC-271908, respectively. Avidin biotin complex (ABC) elite kit (SC-2018) and 3,3-diaminobenzidine (DAB) (SC-216567), were purchased from Santa Cruz Biotechnology, United States. Biotinylated secondary antibody (ab-6789) and DPX mounting media were obtained from Abcam United Kingdom, while Proteinase K (02193981-CF) was purchased from MP Bio United States. All other consumables including Cortisol Enzyme-linked immunoassay (ELISA) kit (Catalog No. 321071403, PerkinElmer Health Sciences, Inc. United States).

### 2.3 Experimental design

#### 2.3.1 Acute epilepsy model and dose optimization

For an acute model of epilepsy, 40 mice were randomly apportioned into six groups mentioned in Table 1 (n = 10). The maximum effectivedose was selected for further chronic epilepsy models.

**TABLE 1:** Study design for acute epilepsy model.

Groups	Treatment received
Vehicle control	2% DMSO in 0.9% normal saline
PTZ group	PTZ 90 mg/kg intraperitoneally (i.p)
Diazepam group	diazepam 2 mg/kg i.p then PTZ 90 mg/kg i.p after 30 min
CHR-0.1	chrysophanol 0.1 mg/kg i.p then PTZ 90 mg/kg ip
CHR-1	chrysophanol 1 mg/kg i.p then PTZ 90 mg/kg ip
CHR-10	chrysophanol 10 mg/kg i.p then PTZ 90 mg/kg ip

#### 2.3.2 Chronic epilepsy model

**FIGURE 1:**
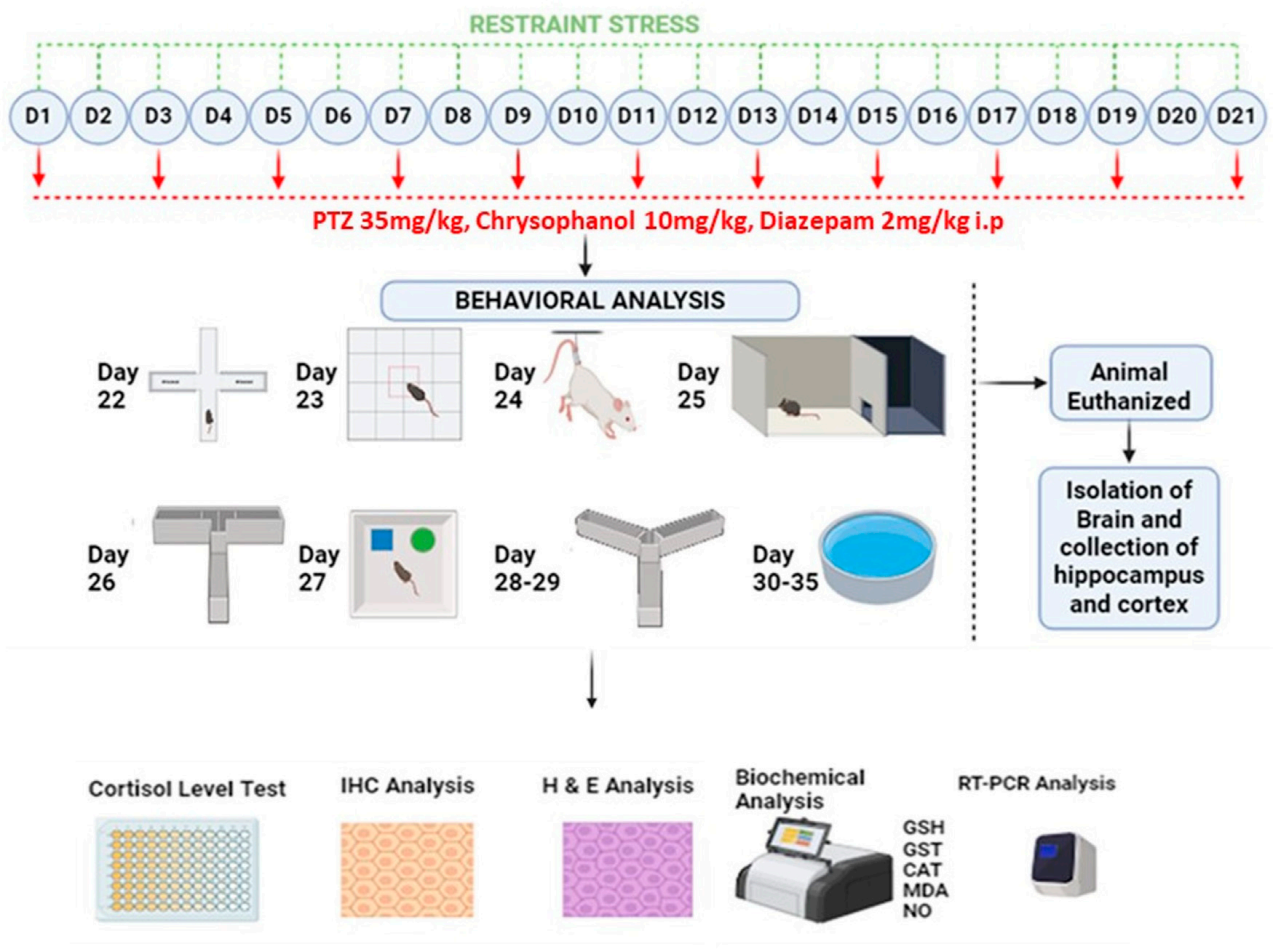
Schematic representation of study design.

Following 1 week of familiarization, 80 mice were randomly assigned into eight groups. Detail of study design is shown diagrammatically in Figure 1 and all experimental groups are shown in Table 2.

**TABLE 2:** Study design for chronic epilepsy model.

Group	Treatment received
Vehicle control	2% DMSO in 0.9% normal saline
Restrain stress (RS)	2h of restrain stress every day for 21 days
Pentylenetetrazole (PTZ)	PTZ 35 mg/kg i.p on alternate days for 21 days
RS-PTZ	2 h of restrain stress + PTZ 35 mg/kg i.p
RS-DIA-PTZ	2 h of restrain stress + diazepam 2 mg/kg + PTZ 35 mg/kg ip
RS-CHR-PTZ	2 h of restrain stress + chrysophanol 10 mg/kg + PTZ 35 mg/kg ip
DIA-PTZ	Diazepam 2 mg/kg + PTZ 35 mg/kg ip
CHR-PTZ	Chrysophanol 10 mg/kg + PTZ 35 mg/kg ip

All groups received their respective treatments for 21 days and were then evaluated for behavioral parameters up to 35 days. Mice in all stress-related groups underwent stress for 2 h each day. On alternative days, PTZ 35 mg/kg was given to mice to induce epilepsy 30 min after restrained stress. Diazepam and chrysophanol were given 30 min before the induction of stress and PTZ.

#### 2.3.3 Induction of stress

For 21 days, mice in 4 groups (RS group, RS-PTZ group, RS-DIA-PTZ group, and RS-CHR-PTZ group) were subjected to 2 h of restraint stress every day. The vehicle control group and the PTZ group did not undergo any restraint stress during this time. To induce stress in mice, the restraint stress technique was used following a previously reported protocol (Rehman et al., 2022; Seewoo et al., 2022). The recommended protocol involves using a restrainer. In this case, 50 mL syringes made of polyvinyl chloride, ventilated from the front with small holes (1.5–2.0 mm), were used as restrainers. The mice were securely held in the restrainer with free movement of their necks for 120 min per day. After this period, the mice were released from their restrainers and returned to their home cages.

#### 2.3.4 Epileptogenesis assessment

Epileptogenesis was evaluated by measuring seizure score given by using Racine’s scale mentioned in Table 3 according to already established protocol (Romariz et al., 2023). Epileptogenesis was induced by pentylenetetrazole 35 mg/kg given through the i.p route. Right after the administration of PTZ, mice were observed for seizure scores for 30 min in all groups.

**TABLE 3:** Racine’s scale for seizure scoring.

Score	Seizure intensity
0	No response
1	Restlessness, hyperactivity, and twitching of vibrissae
2	Head clonus, head nodding, and myoclonic jerks
3	Bilateral or unilateral limb clonus
4	Forelimb clonic seizures
5	Generalized tonic-clonic seizures with the animal falling on its side
6	Hind limb extension
7	Death

### 2.4 Behavioral assessment

#### 2.4.1 Behavioral assessment for stress and memory impairment

##### 2.4.1.1 Elevated plus maze (EPM)

The EPM is a widely acclaimed behavioral test for effectively evaluating stress and anxiety. The wooden maze has two open and two closed arms. Mice were placed in the center of the EPM and given exactly 5 min to explore the maze. The EPM apparatus unequivocally consists of two open arms (15 cm long and 5 cm wide) and two closed arms (also 15 cm long and 5 cm wide). The time spent by mice in both arms of the maze was recorded using a top-mounted camera to evaluate their stress behavior (Gould et al., 2009; Parveen et al., 2023).

##### 2.4.1.2 Open field test (OFT)

OFT provides insight into the stress behavior and locomotor activity of mice. A square wooden apparatus with dimensions of 72 cm × 72 cm x 15 cm was divided into 4 × 4 squares, each with an area of 18 cm × 18 cm. The mice were placed in the center of the field and allowed to move for 5 min. The time spent in the paradigm and total movable time were used to analyze the stress behavior (Malik et al., 2023; Bouguiyoud et al., 2022).

##### 2.4.1.3 Tail suspension test (TST)

The mice were carefully suspended 50 cm above the floor in a tail suspension box with specific dimensions using adhesive tape over the tip of the tail. To ensure accurate results, a 4 cm high and 1 cm wide plastic cylinder was used to prevent climbing during the test. The time spent immobile was then precisely measured by video recording for 6 min on day 24 (Rehman et al., 2022).

##### 2.4.1.4 Light dark box (LDB)

The total time spent in LDB was evaluated by following an already set protocol (Kraeuter et al., 2019; Malik et al., 2023). In a setup, involving a tow-compartment box with a light chamber (21 × 21 × 25 cm) and a dark chamber (20 × 40 × 40) connected by a small opening (7 × 6 cm). On day 25, mice from each group were put in the light compartment of the box with their heads facing the wall of the chamber and allowed to stay there for 5 min. The time spent in the dark slot will definitively indicate the stress behavior in mice.

##### 2.4.1.5 Y-maze test

The Y-maze test is used to analyze memory in rodents. The experiment used a Y-shaped maze with three identical arms labeled A, B, and C (each arm measuring 40 × 8 × 15 cm) positioned at 120°. Mice explore the maze for 8 min in a trial session separated by 24-h intervals for the main test on day 26 while being recorded by a video camera. The recordings are later analyzed for further insights (Lueptow, 2017).

##### 2.4.1.6 Novel object recognition test (NOR)

This experiment explores the rodents’ natural curiosity for the new environment as they explore the novel objects more. Their memory was assessed by testing their ability to distinguish between familiar and novel objects. Each mouse underwent two sessions in a square area (40 × 40 cm) with (38 cm) high walls. During the training session, two identical objects were placed in the area, and mice were given 10 min to explore them. However, in the test session, one object was replaced with a new object, and mouse interaction with both objects was observed. Data was used to calculate the discrimination index with high values indicating improved thinking and memory (Lueptow, 2017; Malik et al., 2023).

$$\text{Discrimination Index}=\left(\text{Novel object duration }-\text{Familiar object duration}\right)÷\left(\text{Novel object duration}+\text{Familiar object duration}\right)$$

##### 2.4.1.7 T-maze test

Spatial memory was evaluated by observing percentage spontaneous alteration (%SAB) in a wooden T-maze set-up that was elevated 60 cm above the ground. The apparatus consisted of arms that were 35 cm long and 7 cm wide, with 15 cm high walls to prevent the animals from falling. The T-maze had a start arm and a goal arm (left and right). A mouse was placed in the start arm and allowed to choose between the right and left arm. After making a choice, the mouse was returned to the start arm for the next trial. A camera mounted on the top was used to record the behavior of the animal during the test. In the T-maze test, if the mouse visited the same arm that it explored in the previous trial, it was considered a perseveration (error), indicating a failure to alternate. On the other hand, choosing the opposite arm was considered a correct alternation, which indicated the animal’s ability to alternate between choices (Mir et al., 2024, D’isa et al., 2021). This behavior was used to assess working memory, where higher rates of alternation indicated better cognitive function, while repeated visits to the same arm suggested impaired memory or cognitive rigidity (Javaid et al., 2023).

$$\%\text{ SAB}=\left[\left(\text{No}\cdot \text{of alterations}\right)/\text{Total arm entries}‐2)\right]\times 100$$

##### 2.4.1.8 Morris water maze test (MWM)

MWM is utilized to measure cognitive deficits and memory impairment in mice. The apparatus consists of a circular pool that is 50 cm in height and 120 cm in diameter. The pool is segmented into four quadrants, and among them, one quadrant serves as the target area. The target quadrant features an elevated platform where the probe is carefully positioned. The probe is submerged 1 cm beneath the water’s surface and is consistently kept at a temperature of 25°C for the duration of the experiment. The platform position is kept constant during the entire experiment (Moron et al., 1979).

###### 2.4.1.8.1 Training and acclimatization phase

The mice underwent a 3-day training session. On the first day, they had 5 min to adopt the pool setting. On the second day, they were positioned on the quadrant with a visible platform for a single trail to familiarize themselves with the platform. On the third day, they were trained with a hidden platform, located 1 cm below the water surface. The platform position during the training session varied from its position in the trial setting.

###### 2.4.1.8.2 Trials

During the trial phase, the mice were assessed on previously learned behavior. The mice had 60 s to find the hidden platform in each trail. In case they could not find it, they were guided to it and allowed to stay on it for 15 s. After each trial mice returned to their home cages for 10 min rest period before starting the new trial.

###### 2.4.1.8.3 Probe trial

On the test day, a probe trial was conducted to test the memory impairment. Following the same protocol, the total time duration for the platform quadrant and the number of crossing over the platform location were recorded for each mouse (Malik et al., 2023).

### 2.5 Sample collection and tissue processing

Mice were sacrificed on day 35 after completion of the MWM test. Mice were euthanized by intraperitoneal administration of Xylazine & Ketamine in 16, and 60 mg/kg respectively. The blood samples were obtained by intracardiac injection. After collection, the samples were centrifuged at 4,000 rpm for 10 min, and the serum was then stored at −80°C. The brain was excised from each animal and the hippocampus and cortex regions of the brain were separated using sharp blades under an iced box. Some samples were preserved in 10% formalin solution for histology, while the rest of the brain samples were stored at −80°C for homogenization. The cryopreserved brain samples were thawed and then weighed using a digital balance. An equal amount of tissue, i.e., 0.1 g was mixed with 1 mL of phosphate-buffered saline (PBS; pH = 7.4) in a 2-mL round-bottom Eppendorf tube. After homogenization, the mixture was centrifuged at 4°C at 4,000 rpm for 10 min to obtain the supernatant for further antioxidant assays.

### 2.6 Estimation of oxidative stress markers

GSH, GST, CATALASE, MDA, and NO levels were measured in the hippocampus and cortex of the brain.

#### 2.6.1 GSH level

Following the described protocol, the GSH level was evaluated in the hippocampus and cortex (Habig et al., 1974). This is a conjugation reaction between the free thiol of GSH and DTNB that generates a yellowish chromophore. 40 ul of DTNB solution was added in each well of the plate followed by the addition of 6.6 uL of sample and 153 uL of phosphate buffer to maintain the pH at 8. Absorbance was recorded at the wavelength of 412 nm using the spectrophotometer.

#### 2.6.2 GST level

GST level was evaluated in the hippocampus and cortex regions of the brain by following previously reported protocol (Habig et al., 1974). This is based on a conjugation reaction between GST CDNB. Each well-contained 10 uL of 1 mM CDNB, 10 uL of 5 mM of reduced glutathione, 10 uL of sample, and 270 uL of buffer solution. The absorbance was recorded at 340 nm wavelength. using a plate reader.

#### 2.6.3 Catalase level

Catalase activity was analyzed in the hippocampus and cortex by using the prescribed protocol (Bouguiyoud et al., 2022). This reaction follows the decomposition of H2O2 by catalase. Absorbance was taken on 240 nm of wavelength by using a microplate reader. Absorbance is directly proportional to the H2O2 level in tissue.

#### 2.6.4 MDA and NO level

MDA and NO levels were analyzed by using the reported protocols respectively (Ohkawa et al., 1979; Tsikas, 2007). For MDA, 20 µL of ferric chloride was mixed with 200 µL of supernatant and 200 µL of ascorbic acid. After incubation at 37°C for 30 min the 500 µL of TCA and TBA were added. Absorbance was taken at 535 nm. NO level was estimated using the Griess reagent method in tissue homogenate and absorbance was recorded at 540 nm.

### 2.7 Histopathology and immunohistochemistry analysis

For histopathological examination, the expunged brain parts were preserved in a buffered formalin followed by dehydration and fixation in paraffin. Using a rotary microtome 5 um thick section was taken (Leica Biosystems) and stained by hematoxylin and eosin. Slides were scrutinized under the optical light microscope at ×10 magnification. Images were scrutinized for morphological changes and no of survival neurons in the hippocampus and cortex (Khan et al., 2019).

Immunohistochemistry of BAX, BCL-2, and NRF-2 was carried out by the avidin-biotin-peroxidase complex ABC method in the hippocampus and cortex of the mouse. The test was performed by following the previously reported protocol (Khan et al., 2019). The hippocampus and cortex regions were separated from the whole brain and fixed on the adhesive slide and then baked at 65°C for 1 h after the preparation of paraffin-coated slides. For deparaffinization of slides, they were dipped into a xylene solution twice for 10 min and then hydrated using ethanol followed by an antigen retrieval step using protein kinase. Blocking was done by using 5% normal goat serum. Primary antibody was applied on slides for specific markers such as BAX, BCL-2, and NRF-2. After this process, they were left overnight and the next day secondary antibody was applied and placed at room temperature for 2 h. ABC Complex was applied and then stained using a DAB solution. Slides were then observed under a microscope for evaluation of expression.

### 2.8 Quantitative real-time polymerase chain reaction to ascertain the expression of TLR4, NFκB, TNF-α, IL-1β, TrkB, BDNF, VEGF, NRF-2, HO-1, and Caspase-3 in hippocampus and cortex

To measure mRNA level, mice hippocampus and cortex were isolated, and total RNA was extricated by HiPure Total RNA Kit by Magen Biotechnology (Catalogue No. IVD4121).by following the manufacturer’s specifications. The concentration of isolated RNA was measured using a nano-drop light spectrophotometer. After analyzing the purity and adjusting the concentration to 2000 ng, RNA was converted to DNA (cDNA) using ThermoFisrt cDNA kit (HRP013 100T) (ZOKEYO, China). These samples were now used for RT-Pcr for measurement of mRNA expression of glyceraldehyde 3-phosphate dehydrogenase (GADPH, housekeeping gene, Primer: forward 5′–3′: ACTCCA CTCACGGCAAATTCA; reverse 3′–5′: TCTCGCTCCTGG AAGATGGT) (Rehman et al., 2022), (TLR4) Primer: forward 5′–3′: ATG​GCA​TGG​CTT​ACA​CCA​CC; reverse 3′–5′: GAG​GCC​AAT​TTT​GTC​TCC​ACA) (Dong et al., 2022), (NF-κB) Primer: forward 5′–3′: GCC​AGA​CAC​AGA​TGA​TCG​CC; reverse 3′–5′: GTT​TCG​GGT​AGG​CAC​AGC​AA) (Dong et al., 2022), (IL-1β) Primer: forward 5′–3′: TGG​ACC​TTC​CAG​GAT​GAG​GAC​A; reverse 3′–5′: GTT​CAT​CTC​GGA​GCC​TGT​AGT​G) (Javaid et al., 2023), (TNF-α) Primer: forward 5′–3′: CCA​CCA​CGC​TCT​TCT​GTC​TAC; reverse 3′–5′: AGG​GTC​TGG​GCC​ATA​GAA​CT) (Javaid et al., 2023), (TrkB Primer: forward 5′–3′: CCA​CGG​ATG​TTG​CTG​ACC​AAA​G; reverse 3′–5′: GCC​AAA​CTT​GGA​ATG​TCT​CGC​C) (Javaid et al., 2023), (BDNF Primer: forward 5′–3′: GGC​TGA​CAC​TTT​TGA​GCA​CGT​C; reverse 3′–5′: CTC​CAA​AGG​CAC​TTG​ACT​GCT​G) (Javaid et al., 2023), (VEGF Primer: forward 5′–3′: AAT​GAT​GAA​GCC​CTG​GAG​TG; reverse 3′–5′: TTT​CTT​GCG​CTT​TCG​TTT​TT) (Elfving et al., 2010). (Nrf2) Primer: forward 5′–3′: CACATCCAG ACAGACACCAGT; reverse 3′–5′: CTA​CAA​ATG​GGA​ATG​TCT​CTG​C) (Alvi et al., 2021), (HO-1) Primer: forward 5′–3′: CGTGCAGAG AATTCTGAGTTC; reverse 3′–5′: AGACGCTTT ACGTAGTGCTG) (Alvi et al., 2021) and (Caspase-3) Primer: forward 5′–3′: ACA​GTG​GAA​CTG​ACG​ATG​ATA​TG; reverse 3′–5′: TCC​CTT​GAA​TTT​CTC​CAG​GAA​TAG) (Huang et al., 2021). The reaction was carried out using 0.5 ul of cDNA with a total 10 ul reaction mixture using SYBR Select Master Mix (CatNo. 4472903, ZOKEYO, China) on Step One Plus Real-time PCR system for relative quantification of each sample (Applied Biosystems, United States). Then, 2^−ΔΔCT^ was calculated after expression analysis.

### 2.9 Estimation of serum cortisol level by enzyme-linked immunosorbent assay ELISA

The serum cortisol level was determined using an ELISA kit according to the manufacturer’s specifications (Catalog No. 321071403, PerkinElmer Health Sciences, Inc. United States).

### 2.10 Molecular docking

Molecular docking was utilized to determine the binding affinity of chrysophanol with various protein targets such as TLR4, NFκB, IL-1β, TNF-α, COX-2, NRF-2, HO-1, BAX, BCL-2, Caspase-3 TrkB, BDNF and VEGF,. The proteins mentioned earlier were examined using the RCSB database and saved in PDB format. To assess the docking interaction of chrysophanol with these proteins, the AutodockVina tool was employed. The results were expressed in terms of the number of hydrogen bonds and binding energies.

### 2.11 Statistical analysis

The data was analyzed using SPSS software version 25. One-way and two-way ANOVA was used followed by the least significant difference (LSD) test for multiple comparisons. The data was presented as mean ± standard deviation (S.D.), and GraphPad Prism version 9.5.0 was used to create graphs. Levene test for homogeneity is determined to include the impact of variance. The value of Levene test is greater than 0.05.

## 3 Results

### 3.1 Dose optimization and seizure assessment in acute epileptic model

**FIGURE 2:**
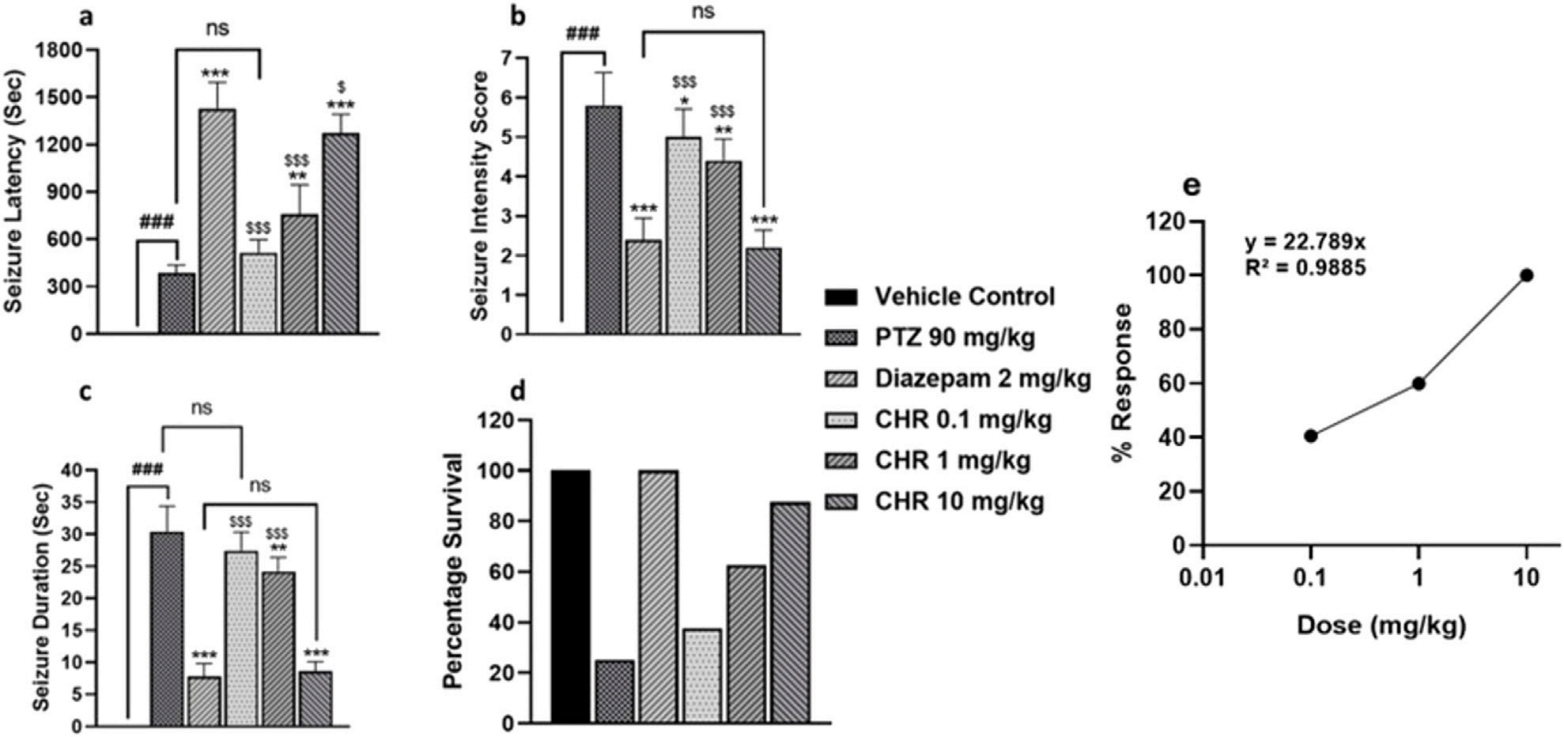
Dose optimization and seizure assessment in acute epileptic model **(A)** seizure latency **(B)** seizure intensity score **(C)** seizure duration **(D)** percentage survival of mice **(E)** Dose Response Curve for chrysophanol. Each value represents the mean ± S.D. (n = 10), analyzed using one-way ANOVA with LSD for multiple comparisons. Significance levels are indicated as follows: #*p* < 0.05, ##*p* < 0.01, ###*p* < 0.001 for comparison with the VC group; *p < 0.05, **p < 0.01, ***p < 0.001 for comparison with the PTZ group; and $*p* < 0.05, $$*p* < 0.01, $$$*p* < 0.001 for comparison with the diazepam group.

Initially, the acute study was performed for dose optimization of chrysophanol. Three different doses of chrysophanol were tested, i.e., 0.1 mg/kg, 1 mg/kg, and 10 mg/kg. Seizure intensity, duration, latency period, and survival percentage were determined. Graphs have shown that there were no seizures in the vehicle control group. PTZ group has a maximum seizure intensity score 6 (F (5,24) = 69.78, *p* = 0.001) duration 30s (F (5,24) = 130.99, *p* = 0.001) and minimum latency period 387s (F (5,24) = 104.7, *p* = 0.001) among all groups except CHR 0.1 mg/kg (*p* = 0.099). The percentage survival of animals in the PTZ group was 25%. Data obtained from chrysophanol treatment have shown that the CHR-0.1 group was least effective in controlling seizure in terms of intensity score (5), duration (27s), latency period (516s), and percentage survival of animals (37.5) and was not statistically significant as compared to the PTZ group. The CHR-1 group was effective and showed significant results in seizure intensity score (4), duration (24s), latency (761s), and percentage survival (62.5%). However, the CHR-10 group has shown maximum efficacy against PTZ-induced epilepsy and showed statistically significant results. Seizure intensity score (2) and duration (9s) were significantly reduced in the CHR-10 group with an increased latency period (1271s). The survival percentage (87.5%) was also maximum for the CHR-10 group and minimum for the PTZ group. The dose-response curve shows the maximum response on chrysophanol 10 mg/kg dose (Figure 2).

### 3.2 Chrysophanol treatment reduced seizure intensity score and frequency while increasing the latency period

**FIGURE 3:**
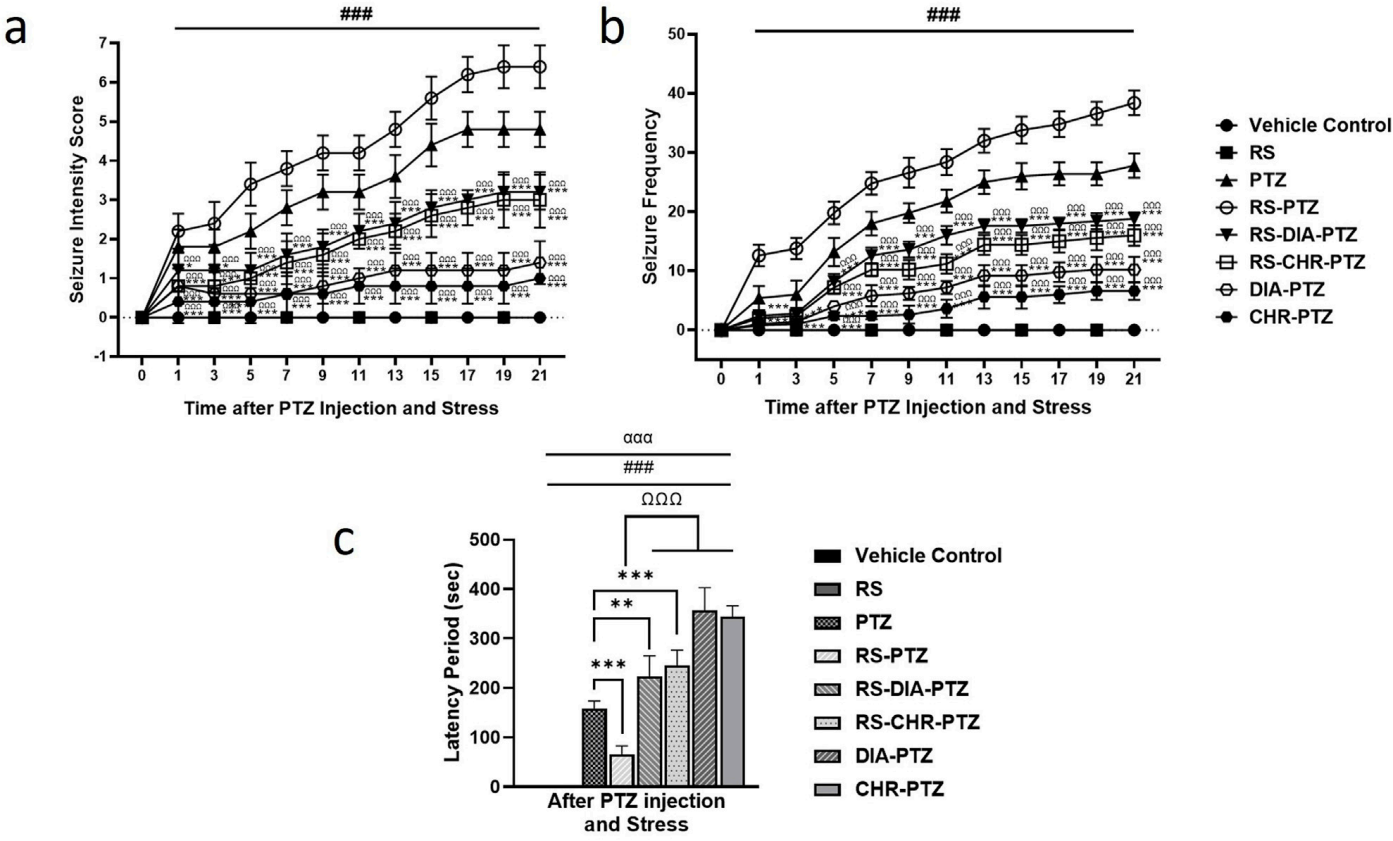
Chrysophanol effectively reduced **(A)** seizure intensity **(B)** seizure frequency, and increase latency period **(C)**. Each value represents the mean ± S.D. (n = 10), analyzed using two-way ANOVA for seizure intensity and frequency and one-way ANOVA for latency period with LSD for multiple comparisons. Significance levels are indicated as follows: ns (non-significant); ^#^ *p* < 0.05, ^##^ *p* < 0.01, ^###^ *p* < 0.001 for comparison with the VC group; *p < 0.05, **p < 0.01, ***p < 0.001 for comparison with the PTZ group; and ^Ω^p < 0.05, ^ΩΩ^p < 0.01, ^ΩΩΩ^p < 0.001 for comparison with the RS-PTZ group.

The vehicle control group and RS group have shown no signs of epileptogenesis. The PTZ kindled group represented increased seizure intensity and frequency and it got worse from day 11 onwards. RS-PTZ group demonstrated the highest score in terms of seizure intensity (F (95,384) = 136.03, *p* < 0.001) and frequency (F (95,384) = 535.29, *p* < 0.001). RS-DIA-PTZ group exhibited a significant reduction (F (95,384) = 136.03, *p* < 0.001) in seizure intensity and frequency than the PTZ group. RS-CHR-PTZ group has shown comparable results with the RS-DIA-PTZ group. It has shown a significant reduction than the PTZ group. The DIA-PTZ group has also significantly reduced the intensity and frequency of the seizure (F (95,384) = 136.03, *p* < 0.001). Meanwhile, the CHR-PTZ group has meticulously decreased the seizure intensity and frequency which is statistically significant. The graph has shown that the RS-PTZ group has a much shorter latency period among all groups. Chrysophanol treatment in RS-CHR-PTZ and CHR-PTZ significantly increased the latency period (F (7,32) = 140.46, *p* < 0.001) Figure 3.

### 3.3 Chrysophanol treatment effectively alleviates stress behavior in mice

**FIGURE 4:**
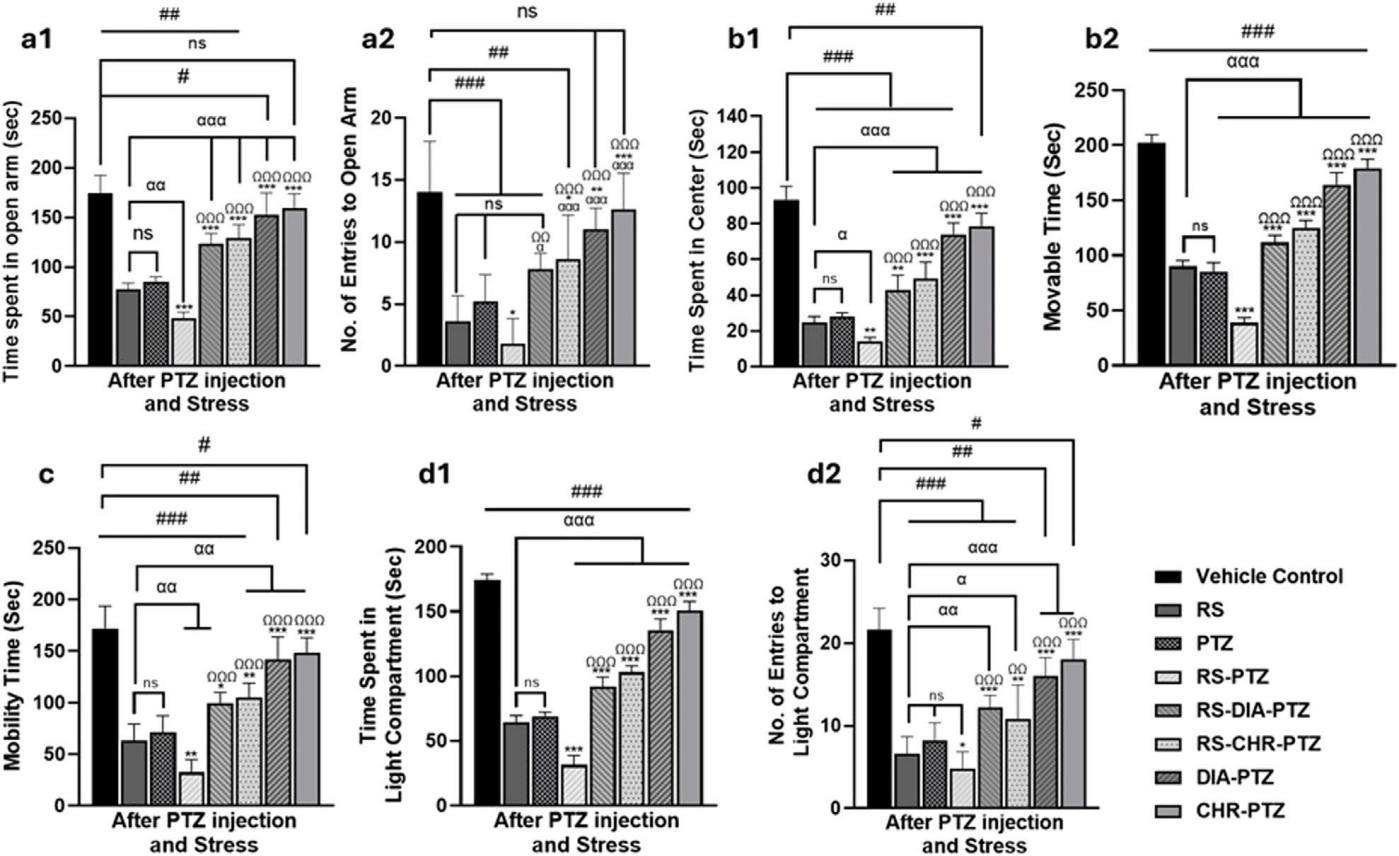
Chrysophanol alleviates stress behavior in mice. Elevated plus maze (a1, a2), Open Field (b1, b2), Tail suspension test (c), Light and dark box (d1, d2). Each value represents the mean ± S.D. (n = 10), analyzed using one-way ANOVA with LSD for multiple comparisons. Significance levels are indicated as follows: ns (non-significant); ^#^ *p* < 0.05, ^##^ *p* < 0.01, ^###^ *p* < 0.001 for comparison with the VC group; ^α^p < 0.05, ^αα^p < 0.01, ^ααα^p < 0.001 for comparison with the RS group; *p < 0.05, **p < 0.01, ***p < 0.001 for comparison with the PTZ group; and ^Ω^p < 0.05, ^ΩΩ^p < 0.01, ^ΩΩΩ^p < 0.001 for comparison with the RS-PTZ group.

The result of the elevated plus maze has shown that the vehicle control group spent the most time in the open arm and had the highest number of entries to the open arm. Compared to the vehicle control group, the RS group spent significantly (F (7,32) = 55.88, *p* < 0.001) less time in the open arm and had no entries (F (7,32) = 13.36, *p* < 0.001). The PTZ group also spent significantly less time in the open arm (F (7,32) = 55.88, *p* < 0.001) and had fewer entries compared to the vehicle control group (F (7,32) = 13.36, *p* < 0.001). The RS-PTZ group has also spent significantly (F (7,32) = 55.88, *p* < 0.001) less time in the open arm with fewer entries. RS + DIA + PTZ group has significantly (F (7,32) = 55.88, *p* < 0.001) improved the time spent in the open arm with an increased no of entries as compared to vehicle control (F (7,32) = 13.36, *p* < 0.001). The RS + CHR + PTZ group has also shown significant (F (7,32) = 55.88, *p* < 0.001) improvement in the time spent in the open arm with an increased number of entries (F (7,32) = 13.36, *p* < 0.001). DIA + PTZ and CHR + PTZ spent comparable time in the open arm with a substantial number of entries to the vehicle control group (Figures 4a1, a2).

On day 23, the vehicle control group spent the most time in the center arena and had the highest moveable time among all other groups in the open field test. RS group significantly (F (7,32) = 102.08, *p* < 0.001) spent less time in the center and decreased movable time (F (7,32) = 264.249, *p* < 0.001) of mice as compared to the vehicle control. PTZ group has also spent significantly (F (7,32) = 102.08, *p* < 0.001) less time in the center arena and movable time (F (7,32) = 264.249, *p* < 0.001) as compared to vehicle control. RS-PTZ group meticulously spent much less time in the center arena (F (7,32) = 102.08, *p* < 0.001) with less movable time (F (7,32) = 264.249, *p* < 0.001) of mice among all other groups. RS-DIA-PTZ group significantly improved the time spent in the center arena with increased moveable time (F (7,32) = 102.08, *p* < 0.001) but markedly less than vehicle control. RS-DIA-PTZ group exhibited statistically significant results in improving the time spent in the central arena (F (7,32) = 102.08, *p* < 0.001)with total movable time (F (7,32) = 264, *p* < 0.001) (F (7, 32) = 249, *p* < 0.001). DIA-PTZ group and CHR-PTZ groups have shown substantial improvement in the total time spent in the arena (F (7,32) = 102.08, *p* < 0.001) and the total movable time of mice (F (7,32) = 264.249, *p* < 0.001) (Figures 4b1, b2).

Mice in the Vehicle control group have shown maximum mobility during TST. RS group has significantly reduced mobility time (F (7,32) = 42.87, *p* < 0.001) as compared to vehicle control. Mobility time in the PTZ group was also significantly (F (7,32) = 42.87, *p* < 0.001) reduced than vehicle control. RS-PTZ group has shown the lowest mobility during TST (F (7,32) = 42.87, *p* < 0.001) compared to vehicle control. RS-DIA-PTZ group significantly improved the mobility time but it was significantly less than vehicle control (F (7,32) = 42.87, *p* < 0.001). mobility time in the RS-CHR-PTZ group has also improved but significantly less than vehicle control. DIA-PTZ and CHR-PTZ groups have shown increased mobility time comparable to vehicle control (F (7,32) = 42.87, *p* < 0.001) (Figure 4c).

Time spent by mice in the light compartment and No. of entries to the light compartment has been determined. The vehicle control group showed a significant preference for the light compartment, making the maximum number of entries. However, mice of RS and PTZ groups have spent significantly (F (7,32) = 292.12, *p* < 0.001) less time in the light compartment as well as No. of entries (F (7,32) = 27.26, *p* < 0.001) to the light compartment as compared to vehicle control. RS-PTZ group exhibited a very short time in the light compartment (F (7,32) = 292.12, *p* < 0.001) and less No. of entries (F (7,32) = 27.26, *p* < 0.001) among all groups. RS-DIA-PTZ group improved the stay of mice in the light compartment (F (7,32) = 292.12, *p* < 0.001) and No. of entries (F (7,32) = 27.26, *p* < 0.001) but has significant differences from vehicle control. RS-CHR-PTZ group has also shown improvement in the time spent in the light compartment and No. of entries. DIA-PTZ and CHR-PTZ groups maximally improved spending time and No. entries in the light compartment. (F (7,32) = 27.26, *p* < 0.001) (Figures 4d1, d2).

### 3.4 Chrysophanol treatment improves memory impairment in mice

**FIGURE 5:**
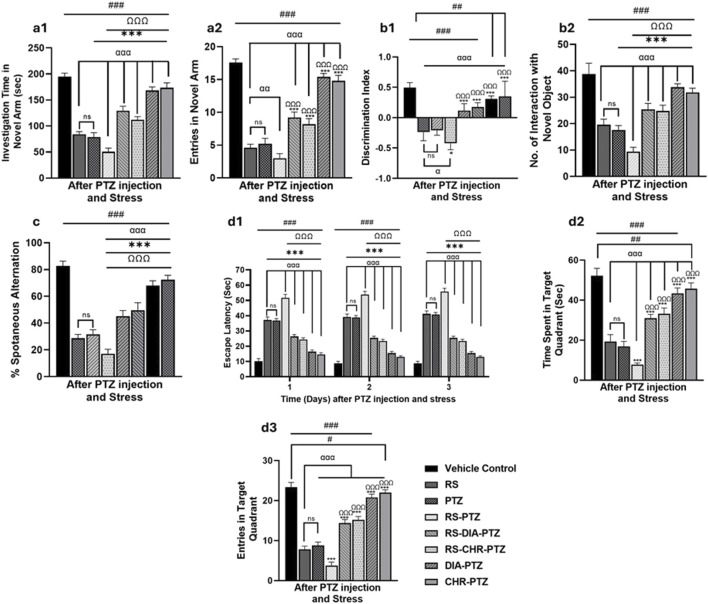
Chrysophanol treatment improves memory impairment in mice. Y-maze (a1, a2), Novel object recognition (b1, b2), T-maze (c), Morris water maze (d1–d3). Each value represents the mean ± S.D. (n = 10), analyzed using one-way ANOVA with LSD for multiple comparisons. Significance levels are indicated as follows: ns (non-significant); ^#^ *p* < 0.05, ^##^ *p* < 0.01, ^###^ *p* < 0.001 for comparison with the VC group; ^α^p < 0.05, ^αα^p < 0.01, ^ααα^p < 0.001 for comparison with the RS group; *p < 0.05, **p < 0.01, ***p < 0.001 for comparison with the PTZ group; and ^Ω^p < 0.05, ^ΩΩ^p < 0.01, ^ΩΩΩ^p < 0.001 for comparison with the RS-PTZ group.

The Y-maze test was conducted to evaluate memory impairment in epileptic mice. The vehicle control group has shown maximum no of entries (F (7,32) = 291.89, *p* < 0.001) in novel arm with maximum investigation time (F (7,32) = 238.31, *p* < 0.001) RS and PTZ groups have significantly fewer entries (F (7,32) = 291.89, *p* < 0.001) with less exploration time in the novel arm (F (7,32) = 238.31, *p* < 0.001) than in vehicle control (*p* < 0.001). Mice in the RS-PTZ group have the least no entries as well as investigation time in the novel arm as compared to vehicle control (F (7,32) = 291.89 and 238.31, *p* < 0.001). Both the RS-DIA-PTZ group and RS-CHR-PTZ group showed significant differences in entries and exploration time compared to the vehicle control (F (7,32) = 291.89 and 238.31, *p* < 0.001). DIA-PTZ and CHR-PTZ groups both have shown maximum improvement in the behavioral paradigm as illustrated in the graph (Figures 5a1, a2).

The careful observation and analysis of the exploration time of mice with familiar and novel objects is a vital step in assessing memory impairment. With meticulous attention to detail, exploration time was recorded and analyzed. Graphs have shown that the vehicle control group has maximum interaction with novel objects and discrimination index at day 27. RS and PTZ groups have significantly less interaction with novel objects (F (7,32) = 89.39, *p* < 0.001) and the least discrimination index (F (7,32) = 33.98, *p* < 0.001) compared to vehicle control. RS-PTZ group has been found to have the least interaction with novel objects (F (7,32) = 89.39, *p* < 0.001)on day 27 and a minimum discrimination index (F (7,32) = 33.98, *p* < 0.001). RS-DIA-PTZ and RS-CHR-PTZ groups were able to significantly improve memory decline and showed significant differences. DIA-PTZ has shown maximum recovery against memory impairment and has a significant difference from the vehicle control group. CHR-PTZ group has also shown comparable results to the vehicle control group (Figures 5b1, b2).

Mice in the vehicle control group have shown maximum %spontaneous alternation in the T Maze test. Nevertheless, RS and PTZ groups have shown a significant decline (F (7,32) = 42.47, *p* < 0.001) in the % spontaneous alternation. RS-PTZ group has shown the lowest percentage which is statistically significant from vehicle control. RS-DIA-PTZ and RS-CHR-PTZ groups have shown significant improvement in overall percent spontaneous alternation (F (7,32) = 42.47, *p* < 0.001). DIA-PTZ and CHR-PTZ groups have exhibited comparable improvement to vehicle control (F (7,32) = 42.47, *p* < 0.001) (Figure 5c).

The impact of restraint stress, along with PTZ, on spatial and learning memory and the effect of chrysophanol was evaluated by the MWM test. During the probe trial, three parameters were measured to evaluate the performance: time spent in the target quadrant, escape latency, and number of crossings over through platform positions. Graphs have shown that the vehicle control group has spent maximum time in the target quadrant with minimum escape latency. The RS and PTZ groups displayed a substantial increase in escape latency (F (23,96) = 512.181, *p* < 0.001) and a decrease in the number of entries (F (7,32) = 344.27, *p* < 0.001) along with a notable reduction in the time spent in the target quadrant when compared to the vehicle control group (F (7,32) = 161.74, *p* < 0.001). The RS-PTZ group spent significantly less time (F (7,32) = 161.74, *p* < 0.001) in the target quadrant and took the longest time to escape (F (23,96) = 512.181, *p* < 0.001) compared to the vehicle control group. In treatment groups, RS-DIA-PTZ and RS-CHR-PTZ groups have improved escape latency (F (23,96) = 512.181, *p* < 0.001) and time spent in the target quadrant. (F (7,32) = 161.74, *p* < 0.001). DIA-PTZ and CHR-PTZ have maximally improved the said parameters comparable to the vehicle control group (Figures 5d1–d3).

### 3.5 Chrysophanol reduced oxidative stress and enhance antioxidant status

**FIGURE 6:**
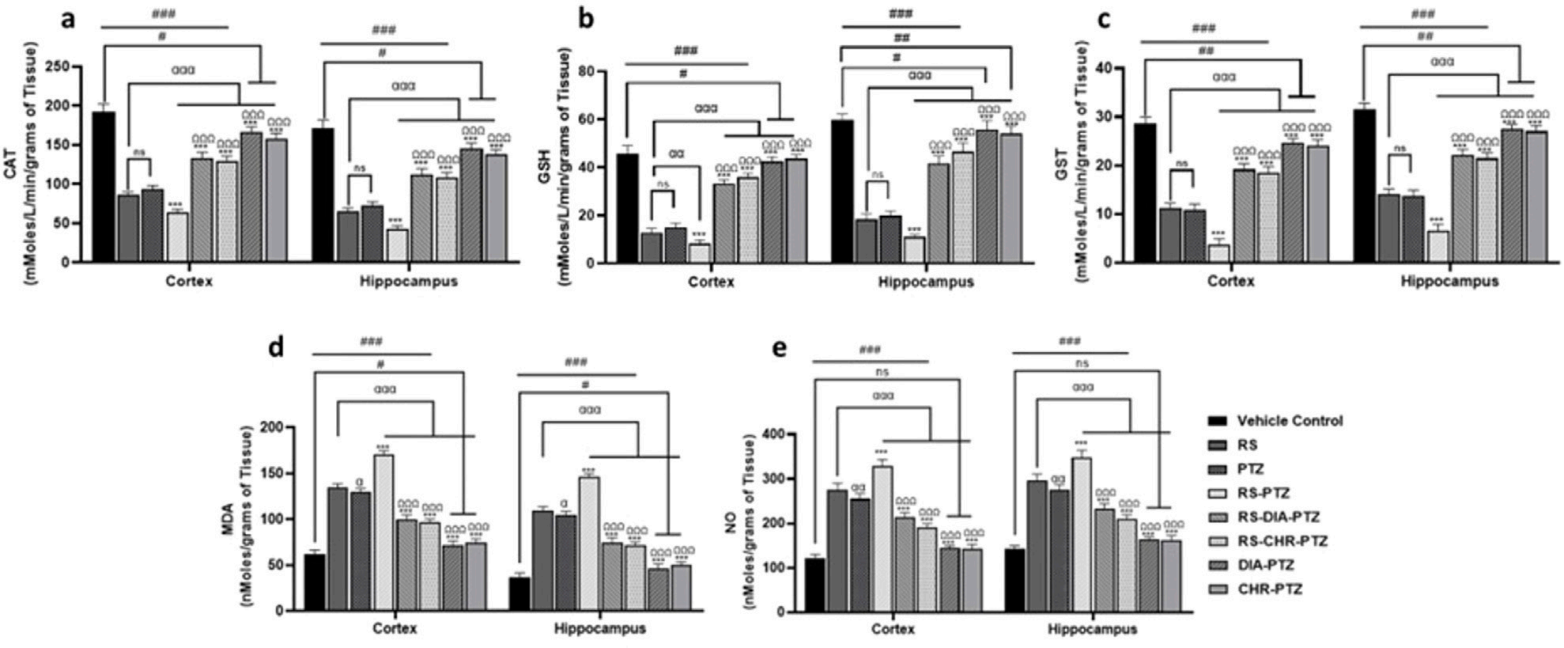
Chrysophanol enhance antioxidant level in hippocampus and cortex **(A)** CAT **(B)** GSH **(C)** GST **(D)** MDA **(E)** NO. Each value represents the mean ± S.D. (n = 10), analyzed using one-way ANOVA with LSD for multiple comparisons. Significance levels are indicated as follows: ns (non-significant); ^#^ *p* < 0.05, ^##^ *p* < 0.01, ^###^ *p* < 0.001 for comparison with the VC group; ^α^p < 0.05, ^αα^p < 0.01, ^ααα^p < 0.001 for comparison with the RS group; *p < 0.05, **p < 0.01, ***p < 0.001 for comparison with the PTZ group; and ^Ω^p < 0.05, ^ΩΩ^p < 0.01, ^ΩΩΩ^p < 0.001 for comparison with the RS-PTZ group.

There was a decreased level of GSH (F (7, 32) = 238.90, *p* < 0.001), (F (7, 32) = 231.66, *p* < 0.001), GST (F (7, 32) = 325.62, *p* < 0.001), CAT (F (7, 32) = 393.32, *p* < 0.001), (F (7, 32) = 317.17, *p* < 0.001) in cortex and hippocampus in all PTZ-kindled mice groups as compared to vehicle control. MDA and NO levels were significantly (F (7, 32) = 350.45, *p* < 0.001), high in PTZ kindled groups as compared to vehicle control. Graphs have shown that chrysophanol was able to significantly improve the anti-oxidant status of both stress-related and non-stress-related PTZ-induced epilepsy. Similarly, MDA and NO levels were significantly decreased after chrysophanol treatment in all groups. RS and PTZ groups have significantly decreased GST, GSH, and CAT levels as compared to vehicle contr. RS-PTZ group has radically decreased anti-oxidant levels and increased oxidant levels among other groups. Chrysophanol treatment groups have been found to significantly improve the anti-oxidant level and decrease the oxidant production as illustrated in the graphs (Figure 6).

### 3.6 Chrysophanol decreased histopathological alterations in neurons

**FIGURE 7:**
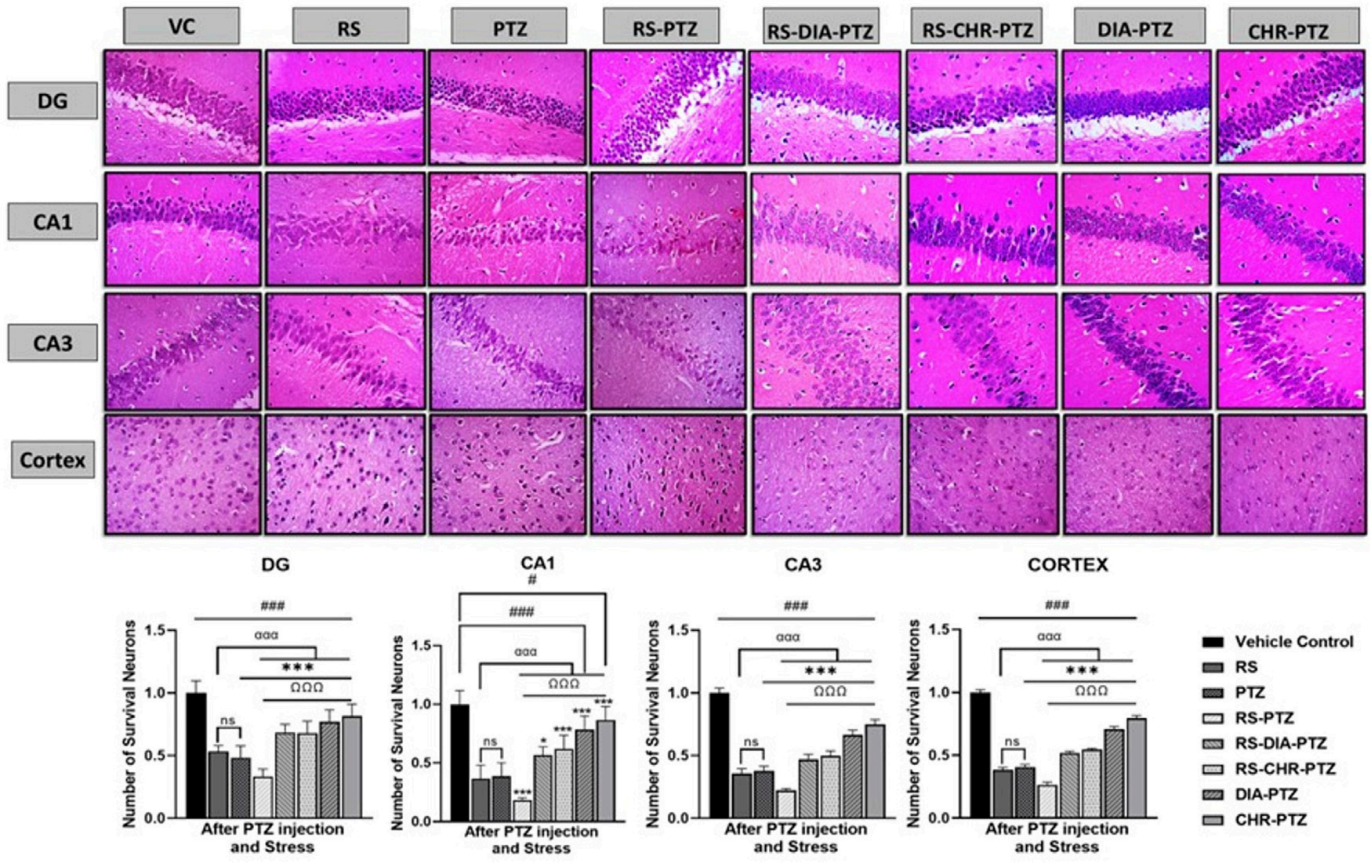
Chrysophanol increases No. of survival neurons in hippocampus and cortex. Each value represents the mean ± S.D. (n = 10), analyzed using one-way ANOVA with LSD for multiple comparisons. Significance levels are indicated as follows: ns (non-significant); ^#^ *p* < 0.05, ^##^ *p* < 0.01, ^###^ *p* < 0.001 for comparison with the VC group; ^α^p < 0.05, ^αα^p < 0.01, ^ααα^p < 0.001 for comparison with the RS group; *p < 0.05, **p < 0.01, ***p < 0.001 for comparison with the PTZ group; and ^Ω^p < 0.05, ^ΩΩ^p < 0.01, ^ΩΩΩ^p < 0.001 for comparison with the RS-PTZ group.

The vehicle control group exhibited no sign of histopathological alterations in the DG, CA1, CA3, and cortex. Histopathological changes have been detected in RS and PTZ groups and no survival neurons were significantly decreased (F (7, 32) = 31.73, *p* < 0.001), (F (7, 32) = 35.43, *p* < 0.001) (F (7, 32) = 227.66, *p* < 0.001), (F (7, 32) = 992.14, *p* < 0.001). RS-PTZ group has shown maximum histopathological variations and reduction in survival neurons as compared to vehicle control. RS-DIA-PTZ group and RS-CHR-PTZ group have significantly overcome the pathological alterations in the hippocampus and cortex. DIA-PTZ and CHR-PTZ have shown the maximum survival rate of neurons among groups as compared to vehicle control (Figure 7).

### 3.7 Effect of chrysophanol on BAX, BCL-2, and Nrf-2 expression

**FIGURE 8:**
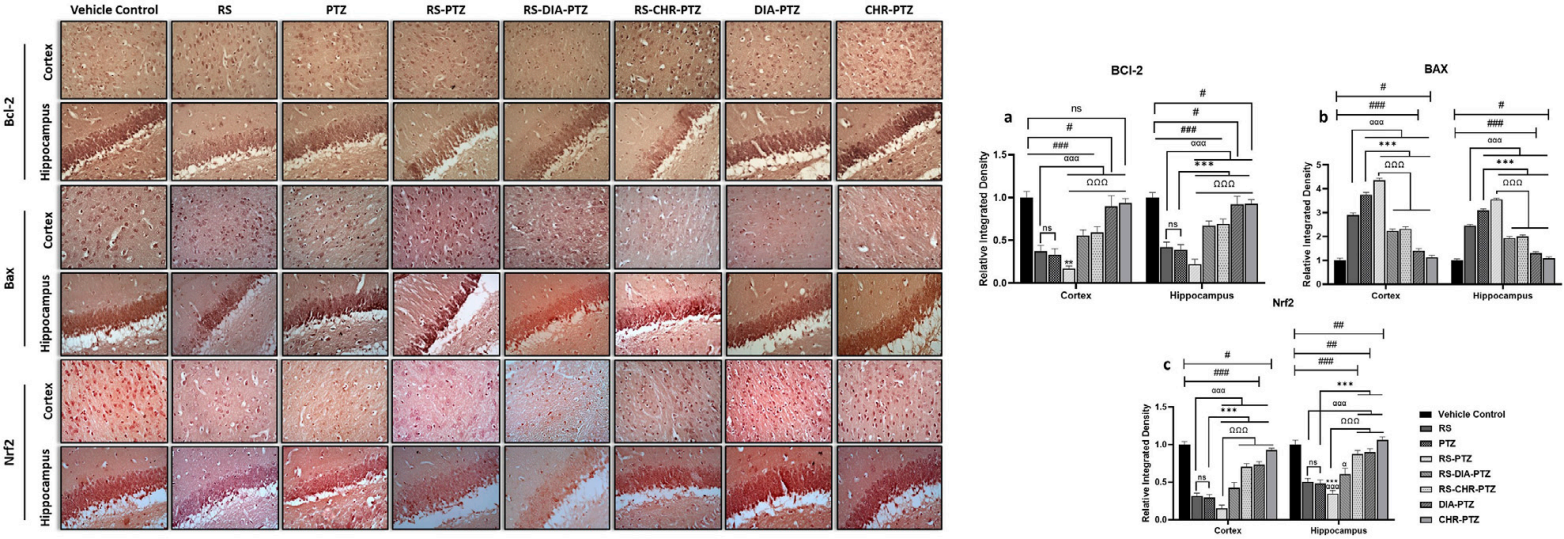
Effect of chrysophanol treatment on Bcl-2, Bax and Nrf2 expression in hippocampus and cortex. **(A)** integrated density of BCL-2 **(B)** integrated density of BAX **(C)** integrated density of Nrf2. Each value represents the mean ± S.D. (n = 10), analyzed using one-way ANOVA with LSD for multiple comparisons. Significance levels are indicated as follows: ns (non-significant); ^#^ *p* < 0.05, ^##^ *p* < 0.01, ^###^ *p* < 0.001 for comparison with the VC group; ^α^p < 0.05, ^αα^p < 0.01, ^ααα^p < 0.001 for comparison with the RS group; *p < 0.05, **p < 0.01, ***p < 0.001 for comparison with the PTZ group; and ^Ω^p < 0.05, ^ΩΩ^p < 0.01, ^ΩΩΩ^p < 0.001 for comparison with the RS-PTZ group.

Immunohistochemistry analysis of hippocampus and cortex sections revealed that BCL-2 and Nrf-2 expressions were remarkably reduced in PTZ-Kindled groups as compared to vehicle control. The highest reduction was seen in the RS-PTZ group and chrysophanol was significantly able to amplify the expression after treatment. In contrast, the expression of BAX was greatly enhanced after PTZ-kindling as compared to vehicle control. RS-PTZ group has shown the highest BAX expression in the hippocampus and cortex. CHR-PTZ group was found to significantly lessen the BAX expression. Figure 8 shows the expression of different markers in the hippocampus and cortex.

### 3.8 Effect of chrysophanol on gene expression in hippocampus and cortex detected through RT/PCR

**FIGURE 9:**
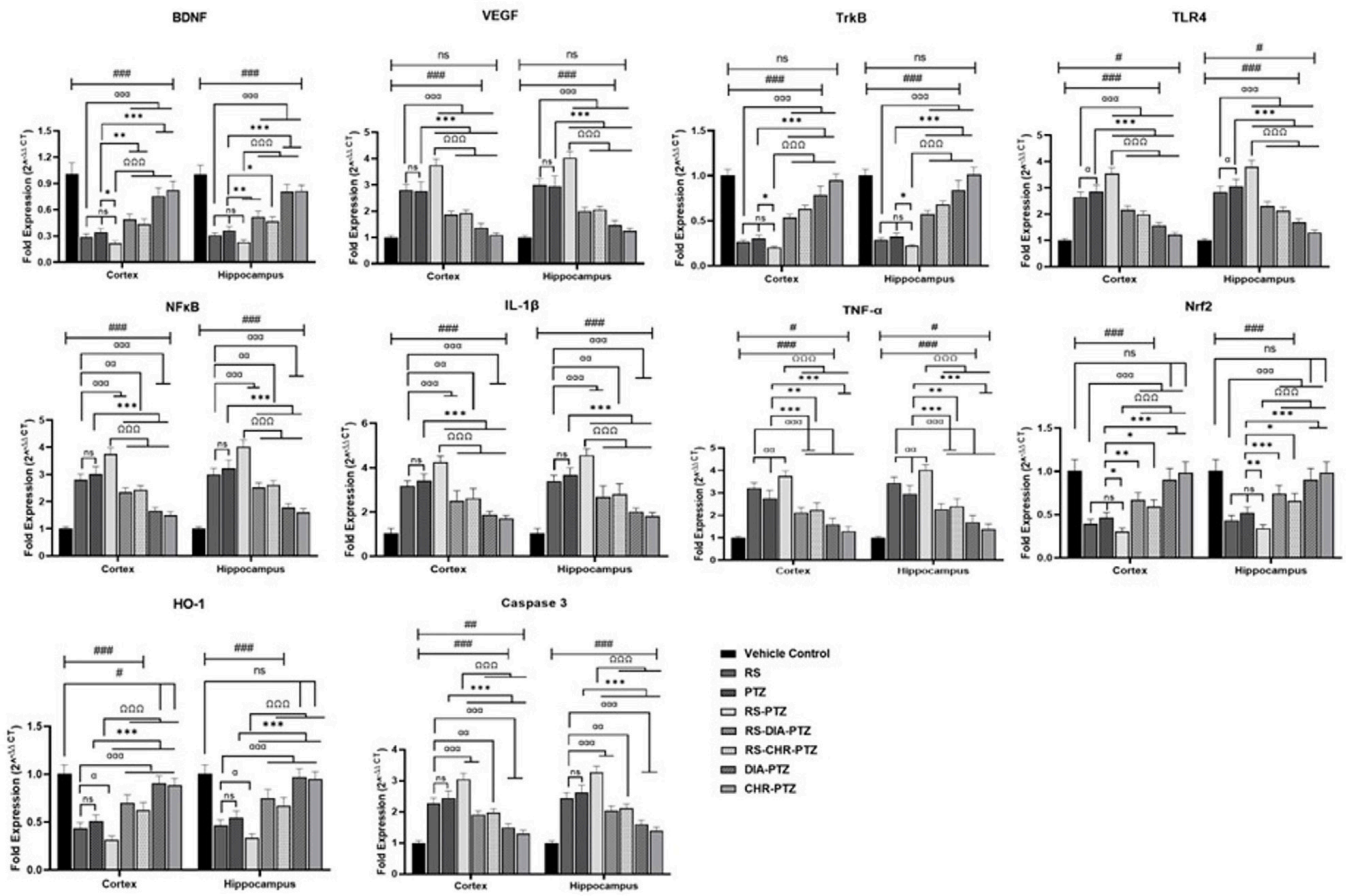
Effect of chrysophanol on mRNA expression of BDNF, VEGF, TrkB, TKR4. NFκB, IL-1β, TNF-α, Nrf2, HO-1, Caspase-3 in hippocampus and cortex. Each value represents the mean ± S.D. (n = 10), analyzed using one-way ANOVA with LSD for multiple comparisons. Significance levels are indicated as follows: ns (non-significant); ^#^ *p* < 0.05, ^##^ *p* < 0.01, ^###^ *p* < 0.001 for comparison with the VC group; ^α^p < 0.05, ^αα^p < 0.01, ^ααα^p < 0.001 for comparison with the RS group; *p < 0.05, **p < 0.01, ***p < 0.001 for comparison with the PTZ group; and ^Ω^p < 0.05, ^ΩΩ^p < 0.01, ^ΩΩΩ^p < 0.001 for comparison with the RS-PTZ group.

mRNA expression of different genes was detected through RT/PCR. The current study revealed that mRNA expression of BDNF, VEGF, TLR4, TrkB, NFκB, IL-1β, TNF-α, Nrf-2, HO-1, and caspases varied significantly among different groups. Figure 9 shows that BDNF level was reduced after PTZ-kindling in stress and non-stressed groups. However, treatment with chrysophanol improved the level in both groups comparable to the positive group. VEGF level was increased after PTZ administration in both RS-PTZ and PTZ groups. Expression altered after chrysophanol treatment in both groups and effectively reduced. Following the trend, mRNA expression of TLR-4, NFκB, TNF-α, IL-1β, and Caspase-3 were evaluated after PTZ-kindling in both RS and RS-PTZ groups. Chrysophanol efficaciously decreased the level in RS and RS-PTZ groups.

### 3.9 Chrysophanol effectively reduced serum cortisol level

**FIGURE 10:**
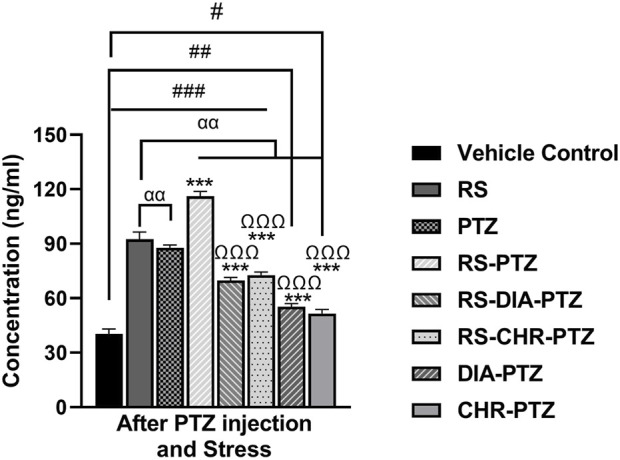
Chrysophanol reduced serum cortisol level in brain. Each value represents the mean ± S.D. (n = 10), analyzed using one-way ANOVA with LSD for multiple comparisons. Significance levels are indicated as follows: ns (non-significant); ^#^ *p* < 0.05, ^##^ *p* < 0.01, ^###^ *p* < 0.001 for comparison with the VC group; ^α^p < 0.05, ^αα^p < 0.01, ^ααα^p < 0.001 for comparison with the RS group; *p < 0.05, **p < 0.01, ***p < 0.001 for comparison with the PTZ group; and ^Ω^p < 0.05, ^ΩΩ^p < 0.01, ^ΩΩΩ^p < 0.001 for comparison with the RS-PTZ group.

ELISA results (Figure 10) have shown that serum cortisol level was maximum in the RS-PTZ group followed by RS and PTZ. Chrysophanol treatment was effectively able to reduce serum cortisol levels. Serum cortisol levels significantly declined in the RS-CHR-PTZ and CHR-PTZ groups after chrysophanol treatment. (F (7, 32) = 600.01, *p* < 0.001).

### 3.10 Molecular docking analysis of chrysophanol

**FIGURE 11:**
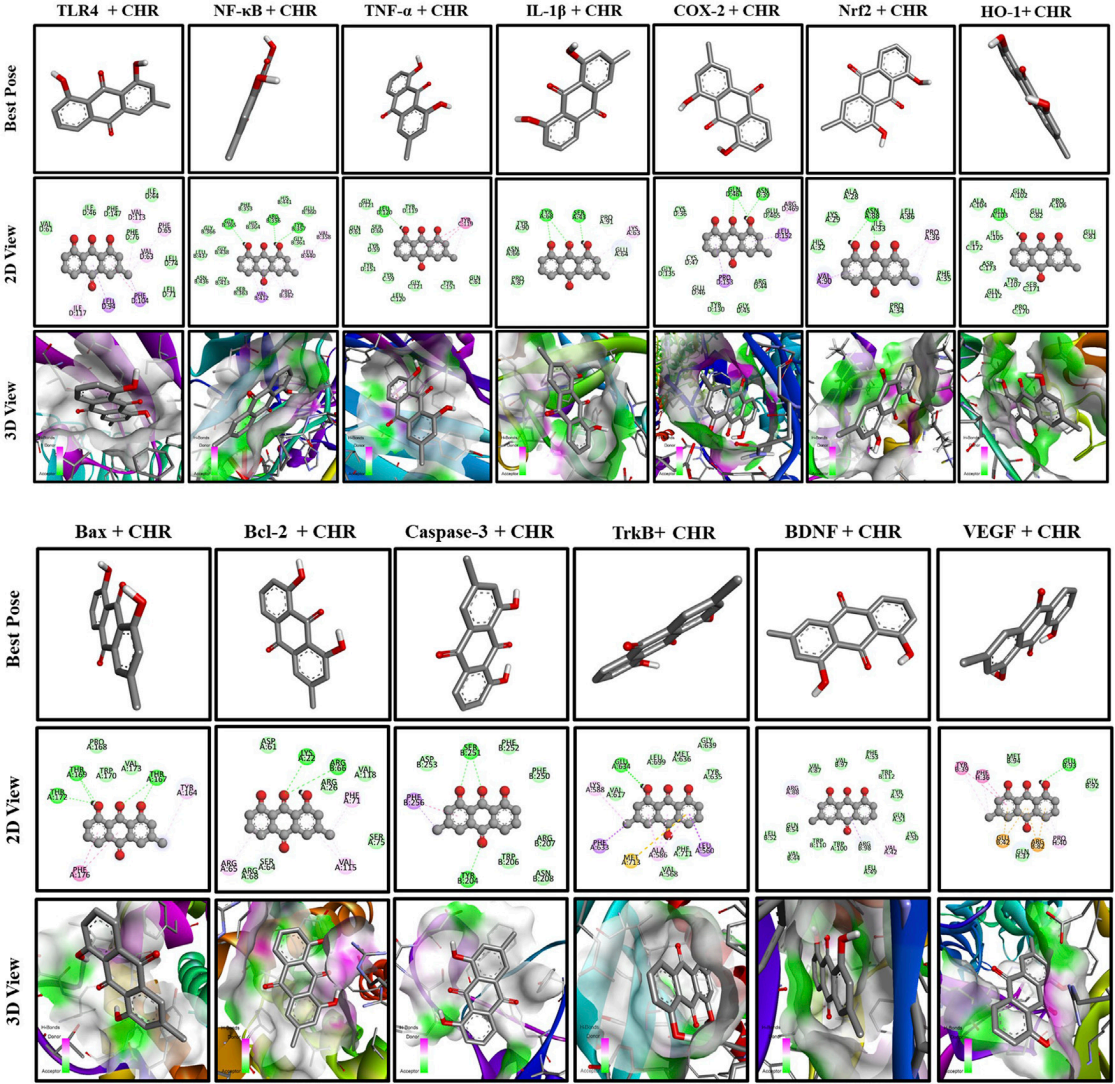
Docking analysis of chrysophanol with proteins interaction including TLR4, NFκB, TNF-α, IL-1β, COX-2, Nrf2, HO-1, BAX, BCL-2, Caspase-3, TrkB, BDNF, VEGF.

To understand the mechanism of interaction between different proteins and chrysophanol, a docking study was carried out. Results of the docking study have shown the interaction of chrysophanol with various protein targets such as VEGF, BDNF, TrkB, Caspase-3, Bcl-2, Bax, HO-1, Nrf-2, COX-2, IL-1β, TNF-α, NFκB, and TLR-4. To explain the effect of these protein interactions with chrysophanol, binding affinities, and binding energies were determined. Binding energies of VEGF (−7.6 kcal/mol**),** BDFN (−9.7 kcal/mol), TrkB (−9.6 kcal/mol), Caspase-3 (−6.5 kcal/mol), Bcl-2 (−7.9 kcal/mol), Bax (-6.3 kcal/mol), HO-1 (−8.0 kcal/mol), Nrf-2 (−6.1 kcal/mol), COX-2 (−9.4 kcal/mol), IL-1β (−6.8 kcal/mol), TNF-α (−8.0 kcal/mol), NFκB (−8.0 kcal/mol), TLR-4 (−8.0 kcal/mol) were observed. Details of binding interactions and binding affinities are given in (Table 4). A 2D and 3D view have been presented in Figure 11.

**TABLE 4:** Molecular docking of chrysophanol.

CHR-protein interaction	PDB ID	Binding affinity (Kcal/mol)	Hydrogen bonds	Hydrogen bond amino acid residue (distance)	Hydrophobic interaction
CHR-TLR4	3VQ2	−8.0	—	—	VAL D:113, PHE D:65, VAL D:63, PHE D:104, LEU D:94, ILE D:117
CHR-NF-κB	1VKX	−8.0	3	GLY B:365 (2.71), ARG B:365 (2.26), TYR B:357 (2.82)	VAL B:358, LEU B:440, PRO B:362, VAL B:412
CHR-TNF-α	2AZ5	−8.0	1	LEU D:120 (2.00)	TYR C:119
CHR-IL-1β	9ILB	−6.8	3	TYR A:68 (2.83, 2.92), SER A:43 (2.04)	LYS A:63
CHR-COX-2	1CX2	−9.4	3	GLN D:461 (6.09, 5.37), ASN D:39 (3.43)	ARG D:469, LEU D:152, PRO D:153
CHR-Nrf2	2FLU	−6.1	2	ASN A:88 (2.45, 2.54)	PRO A:36, VAL A:90
CHR-HO-1	1IRM	−8.0	1	GLU A:103 (2.18)	—
CHR-Bax	5W62	−6.3	5	THR A:172 (2.34), THR A:169 (2.46, 2.79), THR A: 167 (3.00, 2.84)	PHE A:176, TYR A:164
CHR-Bcl-2	2W3L	−7.9	2	LYS A:22 (3.10), ARG B:66 (3.38)	PHE A:71, VAL A:115, ARG A:65
CHR-Caspase-3	2XYG	−6.5	3	SER B:251 (2.91, 2.91), TYR B:204 (2.82)	PHE B:256
CHR-TrkB	4AT4	−9.6	1	GLU A:634 (2.30)	LYS A:588, PHE A:633, ALA A:586, MET A:713, LEU A:560
CHR-BDNF	1BND	−9.7	—	—	VAL A:42, ARG B:98, ARG A:88
CHR-VEGF	2VPF	−7.6	1	GLU B:42 (2.79)	TYR B:39, PHE H:36, PRO H:40, ARG: B:82, GLU B:42

## 4 Discussion

Epilepsy is one of the common disabling neurological conditions characterized by hypersynchronous discharge of neurons in the brain (WHO, 2024; Lang et al., 2018; Stafstrom and Carmant, 2015). Epileptic seizures may also result in unusual sensations, muscular spasms, and loss of consciousness (Stafstrom and Carmant, 2015; Espinosa-Garcia et al., 2021). There are numerous causes of epilepsy including environmental and genetic predispositions (Espinosa-Garcia et al., 2021). Stress is proven to be the widespread cause of epilepsy and one of the common comorbidity (Jhaveri et al., 2023). This study evaluated the neuroprotective effect of chrysophanol in PTZ-induced epilepsy, with stress as the comorbid condition. Overall results of the current study provide remarkable evidence of the ameliorative effect of chrysophanol in stress and without-stress PTZ-induced epilepsy through inhibition of the NFκB pathway and Regulation of VEGF/BDNF. Chrysophanol has successfully reduced the levels of inflammatory cytokines, apoptotic gene expression, and modulation of oxidative stress pathway in PTZ-kindled mice.

Previously reported data have shown that stress exacerbates PTZ-induced seizures with increased intensity and frequency (Khan et al., 2024). Results of this study have also shown that stress has intensified PTZ-induced epilepsy. There was a remarkable reduction in the onset and frequency of epileptic seizures both in stress and without stress-related epilepsy after chrysophanol treatment. Literature reported that Stress and PTZ both are associated with memory impairment and depression-like behavior (Chindo et al., 2024). This study has found similar results, including increased anxiety and memory impairment in both the PTZ group and RS group. Chrysophanol demonstrated significant improvements in the overall anxiety levels and memory status in both simple stressed mice and mice subjected to both stress and PTZ. An overall improvement has been observed in EPM, LDB, OFT, Y-MAZE, NOR, and MWM after chrysophanol treatment. Inflammatory and oxidative stress pathways, both play significant roles in neuroinflammation and neurodegeneration (Teleanu et al., 2022; Li et al., 2013). Oxidative stress is a crucial factor in the pathophysiology of epilepsy and indicates the intrinsic threat of neurodegeneration (Aguiar et al., 2012; Farrell et al., 2017). These pathophysiological changes are accompanied by amplified production of reactive oxygen species (ROS) and weakened capacity of the oxidant scavenging system (Shekh-Ahmad et al., 2019). These free radicals cause the inactivation of different enzymes, lipid peroxidation, and disruption of nucleic acids in the brain (Rowley et al., 2015). It is recognized that PTZ kindling in mice provokes oxidative damage through lipid peroxidation and reduction of anti-oxidant production (Shekh-Ahmad et al., 2019; Łukawski and Czuczwar, 2023). The results of this study follow the already reported data by (Łukawski and Czuczwar, 2023) and Results showed that PTZ-kindled mice with stress and without stress have shown exaggerated production of oxidants such as MDA and NO and depletion of antioxidants including GSH, GST, and CAT resulted in neuronal damage. Chrysophanol lowered the oxidant levels and upgraded the anti-oxidant production in the brain. Reduction in oxidant production and enhancement of anti-oxidant level indicates the neuroprotective effect of chrysophanol in the brain modulating oxidative stress pathway (Xie et al., 2019). Many drugs contribute to epileptic treatment by providing neuroprotection and suppression of oxidative stress pathways (Kośmider et al., 2023; Borowicz-Reutt and Czuczwar, 2020; Cengiz et al., 2005). Unfolding the anti-inflammatory effect of chrysophanol, it is seen that treatment with chrysophanol efficaciously decreased the expression of inflammatory cytokines in the hippocampus and cortex following the NFκB pathway. Various studies have reported that seizures in PTZ Kindled mice cause pathological changes in the brain (Shimada and Yamagata, 2018; Tourov et al., 1996). Histopathological examination of brain tissues of Stressed and without stressed PTZ kindled mice revealed the damage of the granular cell layer in the dentate gyrus and abnormal morphology of the cortex and hippocampus. However, treatment with chrysophanol prevented and helped in the restoration of normal morphology of brain tissues. Immunohistochemistry analysis of the hippocampus and cortex of PTZ-kindled mice clearly showed the enhanced expression of apoptotic genes and reduction in antiapoptotic gene expression, both in stressed and without stressed mice. Nevertheless, chrysophanol magnificently reversed the status and reduced the expression of an apoptotic gene such as BAX and improved the expression of BCL2 and Nrf2 through modulation of the apoptotic pathway (Li et al., 2019). mRNA expression of different genes including VEGF, BDNF, antioxidant genes (Nrf2, HO1), inflammatory (TLR4, NFκB, TNF-α, and IL-1β), and caspases revealed the potential effect of chrysophanol in inflection of various pathways. Studies have reported that BDNF plays an important role in the survival of neurons in the brain, especially in the hippocampus and cortex (Bathina and Das, 2015; Li et al., 2022). Epilepsy has been found to downregulate the level of BDNF in the hippocampus (LaFrance Jr et al., 2010; Karege et al., 2002). Dysregulation of vascular endothelial growth factor VEGF is important in pathogenesis being one of the important mediators of angiogenesis and maintaining the permeability of the blood-brain barrier (Rosenstein et al., 2010). Literature supported the fact that epileptic brains were found to have increased levels of VEGF that alters the vascular permeability and disruption of the blood-brain barrier. mRNA expression has revealed that PTZ-kindled mice have upregulated VEGF expression in the hippocampus. Chrysophanol treatment effectively downregulated the VEGF level. These observations conferred with the reported data that many antiepileptic drugs downregulate the level of VEGF in the brain (Ogaki et al., 2020). Chrysophanol is supposed to modulate the level of these mediators through the BDNF/VEGF pathway. Neuroinflammation and over-expression of inflammatory mediators contribute well to the pathogenesis of epilepsy and the intensity of epileptic seizures. mRNA expression of inflammatory mediators in PTZ kindled mice exhibit upregulation of inflammatory cytokine expression. Stressed mice kindled with PTZ have shown exaggerated expression of these inflammatory mediators. NFκB is the main inflammatory pathway in the production of these inflammatory cytokines that further produce neuroinflammation and neurodegeneration (Shih et al., 2015). Chrysophanol appeared to downregulate the expression of these cytokines verified through mRNA expression. Chrysophanol is hypothesized to inhibit the NFκB pathway and ultimately lead to the inactivation of downstream inflammatory mediators. Reducing the extent of inflammation is one of the key strategies in the treatment of epileptic seizures. neuroinflammation is also related to stress and depression which also contribute to the pathogenesis of epilepsy.

A stressed and depressive brain is more prone to epileptic seizures with high seizure intensity and frequency. All these findings correlate with already reported data (Espinosa-Garcia et al., 2021; Sawyer and Escayg, 2010; Galtrey et al., 2016). Reduction in cortisol concentration after chrysophanol treatment points towards the therapeutic effect of chrysophanol in the reduction of stress. To investigate the direct protein interaction with said targets, molecular docking was carried out against these target proteins such as VEGF, BDNF, Nrf2, HO1, TLR4, NFκB, TNF-α, and IL-1β, BAX, BCL2, and caspases. Results of protein interaction suggest that stable complexes were formed with the proteins and the compound was efficiently docked inside the active site of the target structure. Molecular docking further verifies the affinity of the compound to interact with target proteins. Chrysophanol treatment was not only found effective in suppressing neuronal and oxidant production but also played a significant role in the alleviation of stress-related symptoms in PTZ-kindled mice. Overall results of the current study provide significant evidence of the ameliorative effect of chrysophanol in stress and without-stress PTZ-induced epilepsy through inhibition of the NFκB pathway and Regulation of VEGF/BDNF. Chrysophanol has successfully reduced the expression of inflammatory cytokines, apoptotic genes expression, and modulation of oxidative stress pathway in PTZ-kindled mice. However, it is imperative to use more advanced approaches to explore different mechanistic pathways of chrysophanol activity as well as the gender-based effect of the drug.

## 5 Conclusion

This research has demonstrated that chrysophanol exhibits remarkable neuroprotective activity. It helps to reduce cognitive impairment, neuroinflammation, and oxidative stress by activating TLR4/NFκB, Nrf2/HO-1, and BDNF/VEGF signaling pathways. The study findings support the claim that chrysophanol has anti-epileptic properties and the potential to reduce stress.

## Data Availability

The original contributions presented in the study are included in the article/supplementary material, further inquiries can be directed to the corresponding authors.
